# Effect of mammography screening on stage at breast cancer diagnosis: results from the Korea National Cancer Screening Program

**DOI:** 10.1038/s41598-018-27152-3

**Published:** 2018-06-11

**Authors:** Kui Son Choi, Minjoo Yoon, Seung Hoon Song, Mina Suh, Boyoung Park, Kyu Won Jung, Jae Kwan Jun

**Affiliations:** 1 Graduate School of Cancer Science and Policy, National Cancer Center, 323, Ilsan-ro, Ilsandong-gu, Goyang-si, Gyeonggi-do 10408 Republic of Korea; 2 National Cancer Control Institute, National Cancer Center, 323, Ilsan-ro, Ilsandong-gu, Goyang-si, Gyeonggi-do 10408 Republic of Korea

## Abstract

In Asian countries, breast densities and the proportion of younger women with breast cancer are higher than those in Western countries. This study was designed to determine differences in stage at diagnosis of breast cancer among Korean women according to screening history. The study population was derived from the Korea National Cancer Screening Program (KNCSP). The study cohort comprised 17,689 women who were newly diagnosed with breast cancer in 2011 and were invited to undergo breast cancer screening via the KNCSP between 2002 and 2011. Ductal carcinoma *in situ* (DCIS) and localized breast cancer were most frequent in both ever-screened and never-screened patients. Late stage cancer was significantly more frequent in never-screened patients, compared with ever-screened patients. Compared to never-screened women, the odds ratio (OR) for being diagnosed with early stage breast cancer among screened women was 1.41 (95% Confidence Interval [CI] = 1.28–1.55). The OR for being diagnosed with early stage breast cancer was highest among patients who underwent screening three times or more (aOR = 1.89, 95% CI = 1.57–2.29). Screening by mammography was associated with diagnosis of early stage breast cancer in Korean women. However, significant increases in the diagnosis of DCIS and localized breast cancers among ever-screened patients suggest the possibility of overdiagnosis due to screening.

## Introduction

Breast cancer is a leading cause of cancer death in Korea^1^. The age-standardized rate (ASR) for female breast cancer mortality has increased from 4.2 per 100,000 in 1999 to 5.4 per 100,000 in 2014. Further, the incidence of female breast cancer in Korea has increased constantly (ASR 20.9 per 100,000 in 1999 to 47.7 per 100,000 in 2014), although annual percentage changes (APCs) therein have slowed since 2005^1^. At present, while the incidence of female breast cancer remains low in Korea, compared to that in Western countries, rises in breast cancer incidence and mortality are expected due to trends toward a “Westernized” lifestyle characterized by delayed age at first birth, decreased parity, a diet rich in saturated fats, and a sedentary lifestyle^2–4^.

While mammography is reported to be effective at reducing breast cancer mortality^5–7^ and is widely conducted in many Western countries for breast cancer screening, mammography screening is not common in many Asian countries^8^. To date, only a few Asian countries have introduced mammographic screening as part of organized screening programs^9,10^. In Japan, Korea, and Singapore, organized mammography screening programs were only recently introduced in the early 2000s^11–13^; a high-risk group approach was initiated in Taiwan^14^. In Korea, a nationwide breast cancer screening program was started in 2002 as part of the Korea National Cancer Screening Program (KNCSP), which provides biennial mammography screening for women aged 40 years or over^15^.

Generally, mammography shows lower sensitivity in younger subjects and in individuals with dense breast tissue^16,17^. In Asian countries, breast densities and the proportion of younger women with breast cancer are higher than those in Western countries^18^. Asian women tend to have small and dense breasts, factors known to reduce the diagnostic accuracy of mammography^19–21^. Also, the peak age of breast cancer diagnosis in Asian women (Chinese, Korean, and Japanese) is between 45 and 55 years old, about 10–20 years younger than that in Caucasian women^3^. Thus, we suspect that the effectiveness of mammographic screening in Asian women may differ from that in Western women. A meta-analysis performed by the US Preventive Services Task Force, published in 2009, identified a relative risk reduction in breast cancer mortality of 15% in women aged 39–49 years at randomization who were invited for screening, similar to that for older women; however, a lower absolute reduction was noted and a greater number of women needed to be invited^22^. Recently, a UK Age trial reported a significant reduction in breast cancer mortality in the intervention group, compared with the control group, in the first 10 years after diagnosis (rate ratio, 0.75, 0.58–0.97), but not thereafter (rate ratio 1.02, 0.80–1.30), among tumors diagnosed during the intervention phase. The overall rate ratio for breast cancer mortality was 0.88 (95% CI 0.74–1.04)^23^.

Although a randomized controlled trial is the most ideal study design for evaluating screening effectiveness, no such intervention studies addressing the effectiveness of mammographic screening have been performed in Asian women. Thus, the primary aim of this study was to determine whether stage at diagnosis of breast cancer differed among breast cancer patients who participated in the KNCSP (ever-screened) and women who had no screening history (never-screened) using data from the nationwide breast cancer screening program in Korea. Further, this study aimed to identify potential associations between stage at diagnosis of breast cancer and number of mammography screenings and time interval from the most recent mammography screening.

## Methods

### Study population

**Figure 1:**
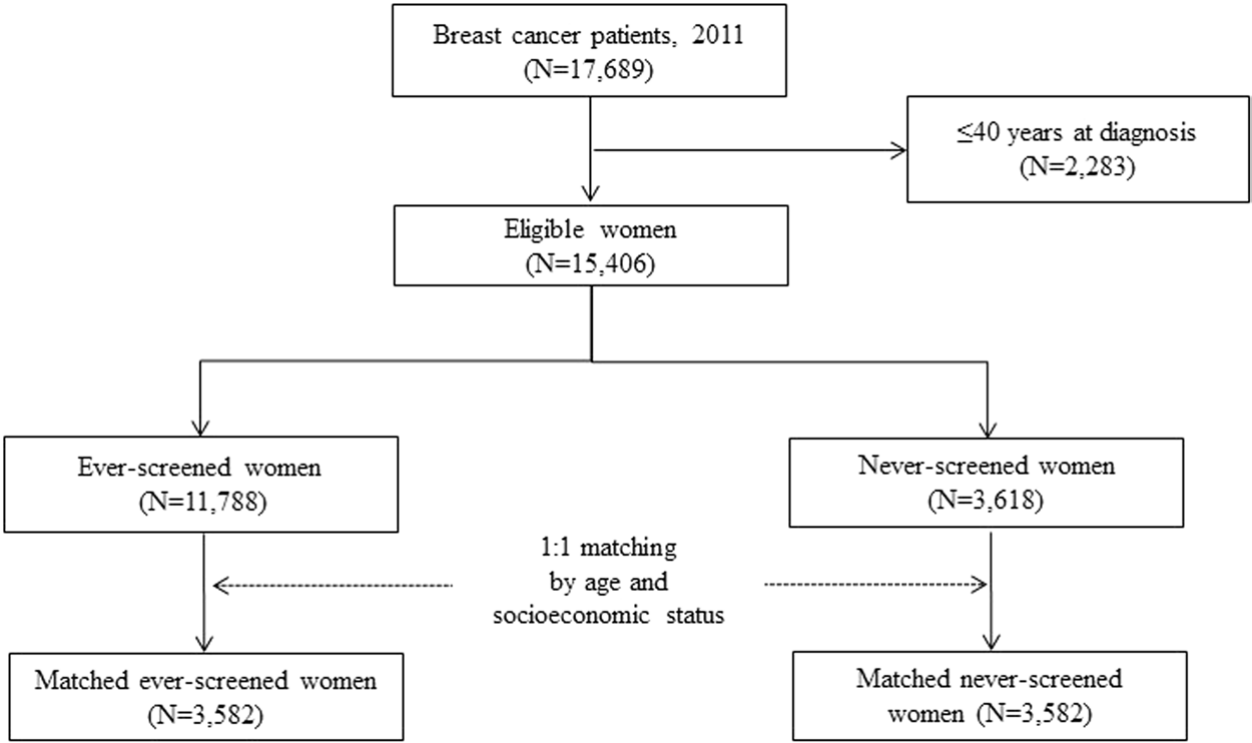
Selection of study sample.

The study cohort comprised women aged 41 years or over who were diagnosed with ductal carcinoma *in situ* (DCIS) or invasive breast cancer in 2011 as registered in the Korean Central Cancer Registry (KCCR)^24^. Even though the KNCSP invites women aged 40 years or over, study subjects were restricted to those 41 years of age or over to ensure that they had at least one occasion on which to receive a breast cancer screening. Among the 17,689 breast cancer patients identified, including both DCIS and invasive breast cancer, 15,406 had been invited to undergo breast cancer screening through the KNCSP in 2002–2011 and were included in the final analysis (Fig. 1).

In the KNCSP, all women aged 40 years or over (no upper age limit) receive a letter biennially from the National Health Insurance Service (NHIS) at the beginning of the year inviting them to undergo a mammographic exam to screen for breast cancer at a clinic or hospital designated as a breast cancer screening unit by the NHIS. In 2015, the Korea National Breast Cancer Screening Recommendation Committee set the upper age limit for mammography screening at 69 years in consideration of the balance between benefits and harms of screening^9^. The committee recommends selective screening mammography in women 70 years or older according to individual risk and preference. However, the KNCSP has not set the upper age limit so far because some women over the age of 69 years still want to undergo breast cancer screening. In 2002, 4,873,996 women were invited to undergo mammography screening. The number of target women continued to rise, and in 2011, a total of 6,889,441 women were invited. Breast cancer screening rates increased from 9.4% in 2002 to 40.5% in 2011.

Mammography imaging results have been reported by the KNCSP using the Breast Imaging Reporting and Data System since 2007. The screening units report mammography results to the NHIS through a web-based database maintained by the NHIS^15^. The overall recall rate of mammography screening during the study period was 14.2%. Participants with a positive screening result on mammography test are recommended to receive a follow-up test (i.e., ultrasonography). However, ultrasonography test is not covered by either the KNCSP or NHIS, and women who undergo a follow-up test must pay for it herself.

The KNCSP database includes the demographic characteristics of all invitees, as well as screening results and written informed consent of those who attend screening. Using this KNCSP database, we classified breast cancer patients according to screening history between January 1, 2002 and December 31, 2011 (11,788 ever-screened and 3,618 never-screened). Since age and socioeconomic status are two of the most powerful factors affecting breast cancer occurrence and death in Korea, never-screened breast cancer patients were matched with the ever-screened breast cancer patients according to age and socioeconomic status. If there was no age match for a given patient, we considered a range of 2 years above or below the target age. A total of 3,582 never-screened breast cancer patients were matched to the same number of ever-screened breast cancer patients.

### Measures

Information on stage at breast cancer diagnosis, anatomic site, and histological classification was obtained from the KCCR. Tumor stage is recorded in the KCCR as localized, regional, distant, or unknown in accordance with the categories used in the Surveillance, Epidemiology, and End Results (SEER) of the National Cancer Institute^25^: Localized tumors are confined entirely to the breast and lack serosal involvement (node negative, no skin or chest wall involvement). Regional tumors refer to a neoplasm that extends beyond the limits of the breast, invading the surrounding tissue (node positive, skin or chest wall involvement). Distant tumors refer to a neoplasm that spreads to parts of the body remote from the primary tumor. Unknown stage is defined as a neoplasm lacking sufficient information with which to assign a stage. Also, ductal carcinoma *in situ* (DCIS) is defined as a neoplasm confined to the duct system without invading the surrounding stroma.

Information on socioeconomic status and screening history was extracted from the KNCSP database. Socioeconomic status was categorized into three groups according to health insurance type: (a) medical aids program (MAP) recipients (extremely poor people who received livelihood assistance and were unable to pay for health care or insurance), (b) NHIS beneficiaries with a premium at 50% or under, and (c) NHIS beneficiaries with a premium above 50%. Further, breast cancer patients were categorized according to number of exams received between 2002 and 2011 (screening frequency: once, twice, three times or more, or never-screened) and intervals between date of breast cancer diagnosis and the preceding screening date (time interval since screening: ≤11 months, 12–23 months, 24–35 months, 36 months or over, or never-screened). Further, the ever-screened group was classified into three groups according to mammography examination results and time interval between screening and breast cancer detection: (a) screen detected cases were defined as breast cancer registered to the KCCR within two years of a positive mammography examination in the KNCSP, (b) interval detected cases were defined as breast cancer registered to the KCCR after a negative mammography screening and before the subsequent scheduled screening mammography in the KNCSP, and (c) non-compliant cases were defined as breast cancer registered to the KCCR at least two years after examination in the KNCSP.

### Statistical analysis

Since the number of never-screened women was too small to compare them with ever-screened women, we conducted statistical analysis for both matched and un-matched datasets. Demographic characteristics, socioeconomic status, anatomic sites, histological classifications, and stages at diagnosis for the ever-screened and never-screened breast cancer patients were compared using χ^2^-tests. Conditional logistic regression was performed to investigate relationships between stage at diagnosis and history of screening in matched ever- and never-screened breast cancer patients. Odds ratios (ORs) for being diagnosed with DCIS and localized breast cancer were estimated. Although conditional logistic regression was conducted separately to include or exclude unknown stage, there was no difference in the results thereof, since cases of unknown stage were few in number. Therefore, only the results from analyses including the cases of unknown stage are presented in this paper. Analyses by age, socioeconomic status, screening frequency, time interval since screening, and manner of breast cancer detection were stratified to investigate which groups would benefit from breast cancer screening. SAS software (ver. 9.1; SAS Institute Inc., Cary, NC, USA) was used for all statistical calculations. The data that support the findings of this study are available from the NHIS and the Ministry of Health and Welfare and were used under license for the current study. Restrictions to their availability apply, and the data are not publicly available. Data are, however, available from the authors upon reasonable request and with permission of the Ministry of Health and Welfare.

### Ethics

This retrospective study was approved by the Institutional Review Board of the National Cancer Center, Korea (IRB No.: NCCNCS08129). With permission from the Ministry of Health and Welfare, we regularly obtained de-identified data from the NHIS, and the need for informed consent for this specific study was waived, since the KNCSP database is quite large. All experiments and methods in this study were performed in accordance with the Declaration of Helsinki.

## Results

### Characteristics of the study population

The baseline demographic and tumor characteristics of the ever-screened and never-screened breast cancer patients from both the matched and un-matched datasets are shown in Table 1. Therein, statistically significant differences were noted in anatomic site, histological classification, and stage at diagnosis between the never- and ever-screened patients. Localized breast cancer and DCIS were significantly more frequent in ever-screened patients, compared with never-screened patients (p < 0.001). The distribution of tumor stages differed significantly between never-screened and ever-screened breast cancer patients (p < 0.001).

**Table 1:** Demographic and tumor characteristics of ever-screened and never-screened breast cancer patients in the National Cancer Screening Program

Characteristics	Matched			Un-matched		
	Never (n = 3,582)	Ever (n = 3,582)	P-value	Never (n = 3,618)	Ever (n = 11,788)	P-value
Age at diagnosis, years			1.00			<0.0001
41–49	1,617 (45.1)	1,617 (45.1)		1,617 (44.7)	4,198 (35.6)	
50–59	1,139 (31.8)	1,139 (31.8)		1,139 (31.5)	4,540 (38.5)	
60–69	437 (12.2)	437 (12.2)		437 (12.1)	2,107 (17.9)	
70≤	389 (10.9)	389 (10.9)		425 (11.8)	943 (8.0)	
Socioeconomic status			1.00			<0.0001
NHIS with premium over 50%	1,944 (54.3)	1,944 (54.3)		1,965 (54.3)	5,881 (49.9)	
NHIS with premium under 50%	1,477 (41.2)	1,477 (41.2)		1,490 (41.2)	5,460 (46.3)	
MAP recipients	161 (4.5)	161 (4.5)		163 (4.5)	447 (3.8)	
Anatomic site			<0.0001			<0.0001
Inner part	477 (13.3)	496 (13.8)		482 (13.3)	1,623 (13.8)	
Outer part	1,114 (31.1)	1,202 (33.6)		1,122 (31.0)	3,950 (33.5)	
Central portion	1,027 (28.7)	1,083 (30.2)		1,034 (28.6)	3,529 (29.9)	
Axillary tail	4 (0.1)	5 (0.1)		4 (0.1)	11 (0.1)	
Unspecified site	897 (25.0)	710 (19.8)		912 (25.2)	2,396 (20.3)	
Missing	63 (1.8)	86 (2.4)		64 (1.8)	279 (2.4)	
Histological subtype			<0.0001			<0.0001
Ductal carcinoma *in situ*	380 (10.6)	501 (13.99)		383 (10.6)	1,667 (14.1)	
Invasive breast cancer	3,202 (89.4)	3,081 (86.01)		3,235 (89.4)	10,121 (85.9)	
SEER Stage^a^			<0.0001			<0.0001
Ductal carcinoma *in situ*	380 (10.6)	501 (14.0)		383 (10.6)	1,667 (14.1)	
Localized	1,677 (46.8)	1,839 (51.3)		1,695 (46.9)	6,133 (52.0)	
Regional	1,124 (31.4)	1,045 (29.2)		1,130 (31.2)	3,346 (28.4)	
Distant	285 (8.0)	93 (2.6)		291 (8.0)	305 (2.6)	
Unknown	116 (3.2)	104 (2.9)		119 (3.3)	337 (2.9)	

Abbreviation: MAP, medical aids program; NHIS, national health insurance.

^a^Stage definitions adapted from the SEER Cancer Statistics Review were applied: localized, a neoplasm confined entirely to the breast without serosal involvement; regional, a neoplasm that extends beyond the limits of the breast and invades the surrounding tissue; distant, a neoplasm that spreads to parts of the body remote from the primary tumor; and unknown, a neoplasm with insufficient or unavailable information to assign a stage.

### Distribution of stages at breast cancer diagnosis

**Figure 2:**
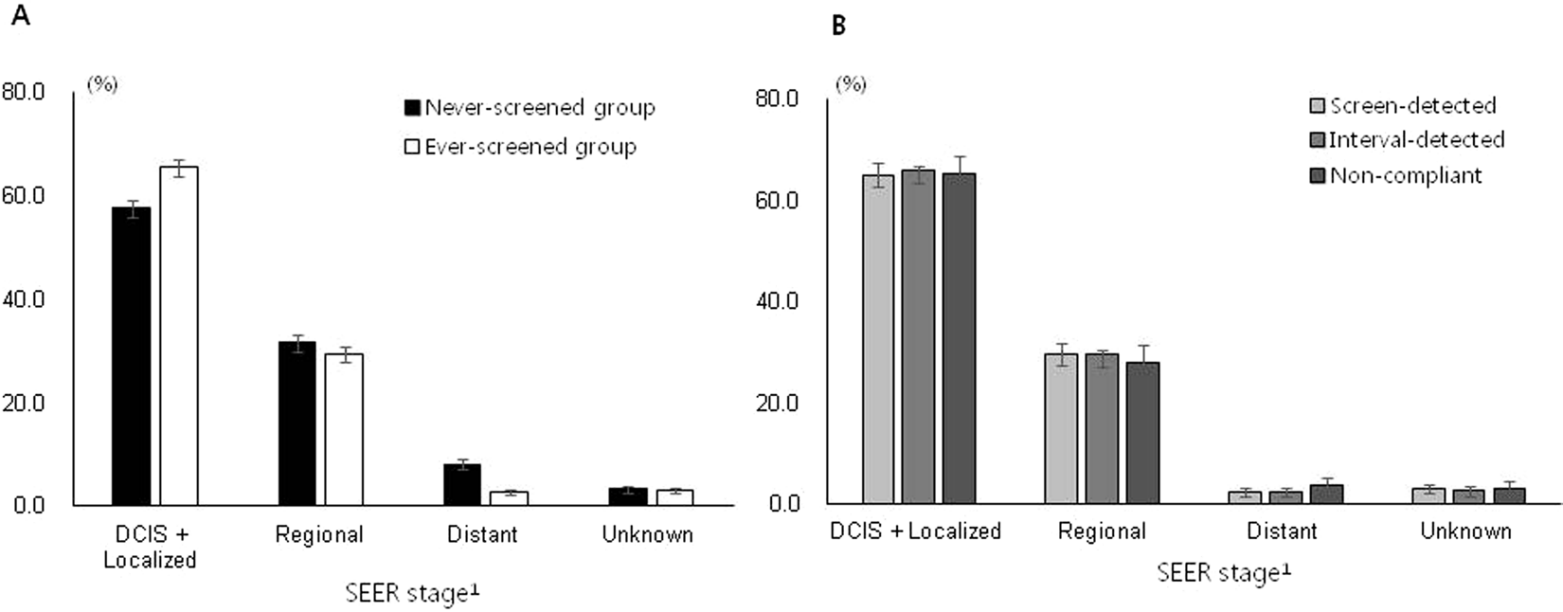
Distribution of stages at breast cancer diagnosis in patients according to history of breast cancer screening in the Korea National Cancer Screening Program. (**A**) Never-screened vs. ever-screened; (**B**) screen-detected, interval, and non-compliant breast cancer among ever-screened women. Abbreviation: DCIS: ductal carcinoma *in situ*; SEER: Surveillance, Epidemiology, and End Results. ^a^Stage definitions adapted from the SEER Cancer Statistics Review were applied: localized, a neoplasm confined entirely to the breast without serosal involvement; regional, a neoplasm that extends beyond the limits of the breast and invades the surrounding tissue; distant, a neoplasm that spreads to parts of the body remote from the primary tumor; and unknown, a neoplasm with insufficient or unavailable information to assign a stage.

Regarding the distribution of stages at breast cancer diagnosis, DCIS and localized breast cancer were the most frequent cancer types in both never-screened and ever-screened patients, although, between these two groups, DCIS and localized cancers were more frequent in the ever-screened patients (Fig. 2A). Regional breast cancer was more frequent in never-screened patients. Among ever-screened patients, distant cancer was more frequent in breast cancers detected more than 2 years after the scheduled breast cancer screening (Fig. 2B). However, there were no significant differences in stage distribution among ever-screened individuals.

### Effect of mammography screening on stage at breast cancer diagnosis

We evaluated the odds of being diagnosed with early stage breast cancer (DCIS and localized breast cancer) versus regional and distant breast cancer, as well as that of unknown stage, according to screening history in the matched and un-matched datasets (Table 2). Overall, ever-screened patients were more likely to be diagnosed with early stage breast cancer than never-screened patients, with statistical significance (adjusted OR [aOR] = 1.41; 95% CI = 1.28–1.55). Specifically, among ever-screened patients, compared with never-screened patients, the OR for being diagnosed with early-stage breast cancer was highest among patients who were aged 70 years or older (aOR = 1.81; 95% CI = 1.33–2.46).

**Table 2:** ORs and 95% CIs for the detection of ductal carcinoma *in situ* and localized breast cancer for ever-screened patients, compared with never-screened patients.

	Matched^a^		Un-matched	
	OR	(95% CI)	OR	(95% CI)
Overall	1.41	(1.28–1.55)	1.46	(1.35–1.57)
Age at cancer diagnosis, years^b^				
41–49	1.34	(1.16–1.55)	1.35	(1.20–1.52)
50–59	1.53	(1.28–1.81)	1.59	(1.39–1.81)
60–69	1.14	(0.87–1.49)	1.35	(1.09–1.66)
70≤	1.81	(1.33–2.46)	1.63	(1.29–2.06)
Socioeconomic status^b^				
NHIS with premium over 50%	1.34	(1.17–1.53)	1.31	(1.18–1.46)
NHIS with premium under 50%	1.50	(1.30–1.74)	1.68	(1.50–1.89)
MAP recipients	1.34	(0.85–2.12)	1.20	(0.83–1.72)
Screening frequency				
Never-screened	1.00	Reference	1.00	Reference
Once	1.27	(1.09–1.47)	1.22	(1.11–1.33)
Twice	1.26	(1.05–1.50)	1.45	(1.31–1.60)
Three times or more	1.89	(1.57–2.29)	1.88	(1.70–2.07)
Time interval since screening, months				
Never-screened	1.00	Reference	1.00	Reference
≤11	1.47	(1.29–1.66)	1.54	(1.41–1.67)
12–23	1.31	(1.06–1.63)	1.37	(1.23–1.53)
24–35	1.61	(1.15–2.26)	1.39	(1.20–1.61)
36 months≤	1.16	(0.86–1.57)	1.20	(1.05–1.38)
Manner of breast cancer detection				
Non-screen detected	1.00	Reference	1.00	Reference
Screen detected	1.43	(1.23–1.66)	1.48	(1.36–1.62)
Interval detected	1.42	(1.21–1.66)	1.52	(1.39–1.67)
Detected after the screening schedule	1.34	(1.07–1.68)	1.28	(1.15–1.43)

Abbreviations: OR = odds ratio; CI = confidence interval; MAP = medical aids program; NHIS = national health insurance service.

^a^Analyses were conducted using conditional logistic regression.

^b^Odds ratio of detecting ductal carcinoma *in situ* and localized breast cancer in screened patients versus never-screened patients in each subgroup.

We further analyzed whether screening frequency was associated with being diagnosed with early-stage breast cancer. Although a significant advantage for screening was observed in all ever-screened patients, the OR for being diagnosed with early-stage breast cancer was highest among patients who underwent screening three times or more during the study period. Further, among ever-screened patients, compared with never-screened patients, the OR for being diagnosed with early-stage breast cancer was no longer significant for a time interval since mammography screening of 36 months or more. Also, compared with never-screened patients, the OR for being diagnosed with early-stage breast cancer was 1.43, 1.42, and 1.34 times higher in the screen-detected, interval-detected, and non-compliant group, respectably.

The results of the analysis with un-matched data were also similar to the matched results, except for results in women aged 60–69 years and for time interval since screening. In the un-matched dataset, ever-screened patients aged 60–69 years were more likely to be diagnosed with early stage breast cancer, and the OR for being diagnosed with early-stage breast cancer remained significant for the time interval since mammography screening of 36 months or longer.

## Discussion

Using population-based data for a large breast cancer screening program in Korea, we investigated the effect of mammography on stages of diagnosis for breast cancer in Korean women. In doing so, we noted a large and significant increase in the diagnosis of DCIS and localized breast cancer, in addition to a corresponding decrease in the diagnosis of regional and distal breast cancer, among ever-screened patients relative to never-screened patients. These data suggest that the implementation of a larger breast cancer screening program in Korea may have improved the detection of early stage breast cancers and decreased the occurrence of late stage breast cancers in Korean women.

Screening, however, may be increasing the burden of low-risk cancers without significantly reducing the burden of more aggressively growing cancers and, therefore, not resulting in the anticipated reduction in cancer mortality (so called overdiagnosis). The incidence of breast cancer in Korea increased after the introduction of screening, and the increase in the relative fraction of early stage cancers has increased, whereas the incidence of regional cancers has not decreased at a commensurate. According to a report from the Korean Breast Cancer Society, the ASR for female breast cancer, including carcinoma *in situ*, increased from 26.1 per 10,000 in 1999 to 63.9 per 10,000 in 2014 (APC was 6.1%)^26^. Specifically, the ASR of invasive breast cancer in particular increased from 1999 to 2014 with an APC of 5.5%, while the ASR of carcinoma *in situ* increased with an APC of 13.5%^26^. The introduction of an optimal screening test should be followed by an increase in the rate of early disease, followed by a decrease in regional disease, while the overall detection rate remains constant^27^. Thus, even though the current study reported significant increases in the diagnosis of DCIS and localized breast cancers among ever-screened patients relative to never-screened patients, we cannot rule out the possibility of overdiagnosis due to screening. However, recently, the APC of ASR of all breast cancers was found to have slightly declined. In particular, the APC of ASR for invasive cancers had slightly declined since 2007 (from 6.7% in 1999–2007 to 4.1% in 2007–2014)^26^. In general, lead time increases breast cancer incidence artificially in a screened population, and this increase is greatest in the early years of the screening program. Thus, it is difficult to separate the lead-time from overdiagnosis in estimating the magnitude of overdiagnosis. Consequently, longer follow-up is required to estimate the magnitude of overdiagnosis and to determine the effectiveness of breast cancer screening program in Korea.

A recent study of mammography for women aged 40–49 years in Austria described significant decreases of 0.72 (95% CI = 0.60–0.86) in tumors of the breast ≥21 mm in size, of 0.27 (95% CI = 0.17–0.46) in metastatic breast cancers, and of 0.83 (95% CI = 0.71–0.96) in advanced breast cancers, each comparing those exposed to screening to those unexposed to screening, respectively^28^. Another study conducted on patients with breast cancer in Norway found that 50% of breast cancers were localized breast cancer in the post-breast cancer screening program era, compared to 48.5% in the pre-program era^29^. Interestingly, in the post-breast cancer screening program era, the study also discovered a substantial survival benefit among women diagnosed with breast cancer who had yet to have been invited to mammography. These findings are compatible with our study and are consistent with the notion that screening by mammography leads to earlier diagnosis of breast cancer. In the current study, patients screened by mammography were 1.41 times more likely to be diagnosed with DCIS and localized breast cancer, compared to those who had not been screened. Also, compared with never-screened patients, the ORs for being diagnosed with DCIS and localized breast cancer were highest for women screened 11 months or less before being diagnosed, and tended to decrease with an increasing time interval.

As stage at diagnosis of breast cancer is well correlated with survival rate, we suspect that early detection of breast cancer by screening may increase patient survival in Korean women. Indeed, several studies have noted a strong relationship between a lower rate of advanced breast cancer cases detected upon initiation of a screening program and future reductions in breast cancer mortality^30–32^. Tabar *et al*. argued that a reduction in advanced-stage disease by 20% or more offers a 28% reduction in mortality, which should correspond to an approximately 40% reduction in mortality in women who actually undergo screening^30^. According to similar calculations comparing women exposed versus unexposed to screening, we estimated that a 59% relative risk reduction in advanced breast cases in Korea could potentially be obtained, followed by a future reduction in breast cancer mortality.

However, this study had several limitations. First, breast cancer screening effectiveness cannot be estimated by cancer stages, since there might be a gap between cancer stage and mortality related to lead-time and length biases. Further, one should anticipate the possibility of overdiagnosis. A significant increase of early stage cancer is an early indicator of a screening effect resulting from either effective screening or overdiagnosis. Unfortunately, in the current study, we were not able to separate the major benefit of screening from the major harm (overdiagnosis of indolent cancers and DCIS). Even though there is sufficient evidence to acknowledge overdiagnosis as a serious harm from population breast cancer screening, the magnitude of overdiagnosis attributed to mammography screening is uncertain and complicated. Thus, the effectiveness of any screening should be evaluated in terms of whether mortality from cancer is actually reduced in the screened population, and therefore, further study is needed to determine whether breast cancer screening is effective in reducing mortality in Korean women.

Second, this study was conducted as an observational study of women who were either exposed or unexposed to screening, and thus, all known problems associated with this type of study design should be considered, especially selection bias, as well as confounding. It is well known that in the observational study, the inherent bias is important to potential baseline differences in the screened and unscreened groups with respect to factors that are associated with the risk for fatal breast cancer^33^. Unfortunately, we did not have information on all these breast risk factors. Nevertheless, to minimize confounding effects, we were able to match the never-screened and ever-screened breast cancer patients according to age and socioeconomic status, which are known to be strong risk indicators for breast cancer incidence and mortality in Korea^34^. Moreover, as argued in the Handbook on Breast Cancer Screening^35^, any bias due to selection for screening would likely be small in organized programs with invitation schemes based on population registries and with high attendance rates: the lifetime breast cancer screening rate in Korean women is 73.5%.

Third, screening history outside of the KNCSP was unclear. Screening history was identified based on lists of individuals who had undergone breast cancer screening from 2002 to 2011 in the KNCSP database. However, opportunistic breast cancer screening using ultrasonography is often performed, especially for those with dense breasts. This factor might have led to underestimation of the magnitude of the observed screening effect.

Finally, we were unable to exclude symptomatic individuals in the present study, since such information is not available in the KNCSP database and since, in general, symptomatic individuals have often been found to participate in cancer screening. Also, since symptomatic individuals are more likely to be diagnosed with advanced-stage breast cancer, the proportion of early-stage diagnoses in the ever-screened group might be underestimated.

Despite these limitations, the main strength of our study is the fact that we were able to link registry and screening data on an individual basis and that, consequently, we could categorize all incident breast cancer cases according to screening attendance. Additional strengths include the cancer registry’s full coverage and the fact that nearly complete data on cancer stage were obtained. In consideration of these strengths and limitations, this study holds the following implications: First is that breast cancer screening using mammography was associated with an earlier stage at breast cancer diagnosis in Asian women aged 40 years or over. Second is that our intermediate outcomes indicate that the introduction of breast cancer screening for the average-risk population will lead to increased survival among breast cancer patients in Korea. However, significant increases in the diagnosis of DCIS and localized breast cancers among ever-screened patients also suggest the possibility of overdiagnosis due to screening. Thus, further study is needed to assess the magnitude of overdiagnosis of mammography screening and to determine whether breast cancer screening is actually effective in reducing mortality.
